# Sex Differences in Human Brain Structure at Birth

**DOI:** 10.1186/s13293-024-00657-5

**Published:** 2024-10-17

**Authors:** Yumnah T. Khan, Alex Tsompanidis, Marcin A. Radecki, Lena Dorfschmidt, APEX Consortium, Yumnah T. Khan, Alex Tsompanidis, Marcin A. Radecki, Deep Adhya, Bonnie Ayeung, Rosie Bamford, Tal Biron-Shental, Graham Burton, Wendy Cowell, Jonathan Davies, Dorothea L. Floris, Alice Franklin, Lidia Gabis, Daniel Geschwind, David M. Greenberg, Yuanjun Gu, Alexandra Havdahl, Alexander Heazell, Rosemary J. Holt, Matthew Hurles, Madeline Lancaster, Michael V. Lombardo, Hilary Martin, Jose Gonzalez Martinez, Jonathan Mill, Mahmoud Musa, Kathy Niakan, Adam Pavlinek, Lucia Dutan Polit, David Rowitch, Jenifer Sakai, Laura Sichlinger, Deepak Srivastava, Florina Uzefovsky, Varun Warrier, Elizabeth M. Weir, Xinhe Zhang, Carrie Allison, Meng-Chuan Lai, Richard A. I. Bethlehem, Simon Baron-Cohen, Topun Austin, John Suckling, Carrie Allison, Meng-Chuan Lai, Richard A. I. Bethlehem, Simon Baron-Cohen

**Affiliations:** 1 Department of Psychiatry, University of Cambridge, Cambridge, CB2 8AH UK; 2 Autism Research Centre, Department of Psychiatry, University of Cambridge, Cambridge, CB2 8AH UK; 3 Social and Affective Neuroscience Group, IMT School for Advanced Studies Lucca, Lucca, Italy; 4 Department of Psychiatry, University of Pennsylvania, Philadelphia, PA 19104 USA; 5 Lifespan Brain Institute, The Children’s Hospital of Philadelphia and Penn Medicine, Philadelphia, PA 19139 USA; 6 Neonatal Intensive Care Unit, Cambridge University Hospitals NHS Foundation Trust, Cambridge, CB2 0QQ UK; 7 Peterborough Foundation NHS Trust, Cambridge, CB2 8SZ UK; 8 Centre for Addiction and Mental Health, Campbell Family Mental Health Research Institute, Toronto, ON Canada; 9 Department of Psychiatry, The Hospital for Sick Children, Toronto, ON Canada; 10 Department of Psychiatry, Temerty Faculty of Medicine, University of Toronto, Toronto, ON Canada; 11 Department of Psychology, Faculty of Arts and Science, University of Toronto, Toronto, ON Canada; 12 Department of Psychiatry, National Taiwan University Hospital and College of Medicine, Taipei, Taiwan; 13 Department of Psychology, University of Cambridge, Cambridge, CB2 3EB UK

**Keywords:** Sex differences, Brain structure, Neonatal brain, Brain development

## Abstract

**Background:**

Sex differences in human brain anatomy have been well-documented, though remain significantly underexplored during early development. The neonatal period is a critical stage for brain development and can provide key insights into the role that prenatal and early postnatal factors play in shaping sex differences in the brain.

**Methods:**

Here, we assessed on-average sex differences in global and regional brain volumes in 514 newborns aged 0–28 days (236 birth-assigned females and 278 birth-assigned males) using data from the developing Human Connectome Project. We also assessed sex-by-age interactions to investigate sex differences in early postnatal brain development.

**Results:**

On average, males had significantly larger intracranial and total brain volumes, even after controlling for birth weight. After controlling for total brain volume, females showed significantly greater total cortical gray matter volumes, whilst males showed greater total white matter volumes. After controlling for total brain volume in regional comparisons, females had significantly increased white matter volumes in the corpus callosum and increased gray matter volumes in the bilateral parahippocampal gyri (posterior parts), left anterior cingulate gyrus, bilateral parietal lobes, and left caudate nucleus. Males had significantly increased gray matter volumes in the right medial and inferior temporal gyrus (posterior part) and right subthalamic nucleus. Effect sizes ranged from small for regional comparisons to large for global comparisons. Significant sex-by-age interactions were noted in the left anterior cingulate gyrus and left superior temporal gyrus (posterior parts).

**Conclusions:**

Our findings demonstrate that sex differences in brain structure are already present at birth and remain comparatively stable during early postnatal development, highlighting an important role of prenatal factors in shaping sex differences in the brain.

**Supplementary Information:**

The online version contains supplementary material available at 10.1186/s13293-024-00657-5.

## Plain Language Summary

Sex differences in the human brain have attracted substantial scientific and societal interest, but less is known about whether the brain shows sex differences at birth. Studying sex differences at birth can help to understand how prenatal factors (e.g., hormone levels before birth) and early postnatal factors (e.g., exposure to the sensory environment and caregiver interactions) contribute to shaping sex differences in the brain. In this study, we investigated on-average sex differences in brain structure in a large sample of newborn infants shortly after birth. Our findings show that several on-average differences are present at birth, suggesting that factors before birth play an important role in initiating sex differences in the brain.

**Supplementary Information:**

The online version contains supplementary material available at 10.1186/s13293-024-00657-5.

## Highlights

- At birth, males on average show significantly increased total brain volumes compared to females even after accounting for sex differences in birth weight.
- After controlling for total brain volume, females on average show significantly increased total cortical gray matter volumes, while males show increased total white matter volumes.
- After controlling for total brain volume, significant on-average sex differences are observed in regions such as the corpus callosum (F > M), bilateral parietal lobes (F > M), left anterior cingulate gyrus (F > M), left caudate nucleus (F > M), and right medial and inferior temporal gyri (M > F).
- Fewer global and regional volumes showed significant sex-by-age interactions, except for the left anterior cingulate gyrus (F > M) and left superior temporal gyrus (M > F).
- Several sex differences that have previously been observed later in development are present from birth, emphasising the key role that prenatal factors play in initiating sex differences in the brain.

**Supplementary Information:**

The online version contains supplementary material available at 10.1186/s13293-024-00657-5.

## Background

Note on terminology: Throughout this paper, all references to “sex differences” or “on-average sex differences” are intended to reflect differences observed in group averages and not individual cases.

While sex differences in human brain anatomy are well-evidenced (for a meta-analysis, see [1]), their magnitude, significance, and implications remain a matter of substantial ongoing debate (for recent discussions, see [2, 3]). Most notably, their underlying causes are a central point of scientific discussion and remain poorly understood. This area of research is of high importance because the prevalence of various psychiatric, neurological, and neurodevelopmental conditions differs by biological sex [4, 5]. Given that variations in brain development are implicated in these conditions and overlap with neurobiological sex differences, it is likely that sex differences play a key role in the development of these conditions [4, 6, 7]. A better understanding of sex differences, their underlying causes, and their onset could therefore help tailor diagnostic, prognostic, and support strategies to facilitate optimal health outcomes.

Sex differences in brain structure are hypothesised to arise from a complex interplay between multiple biological and environmental factors regulating brain development [8]. The perinatal period is marked by key events that can influence observed sex differences in the brain, and the highly dynamic and malleable nature of brain development during this period can make the brain particularly sensitive to these influences. For instance, during the first and second trimesters of pregnancy, male fetuses produce around 2.5 times more testosterone than female fetuses [9]. This prenatal surge in testosterone is understood as a key early biological mechanism instigating the sexual differentiation of the body and brain [10]. Prenatal factors such as maternal nutrition and toxin exposure are also known to impact fetal brain development [11], potentially in sex-specific ways [12]. Studying sex differences at birth can provide insights into the influence that these prenatal factors hold in shaping sex differences in the brain. After birth, numerous early postnatal factors begin to act on brain development. For instance, gender socialisation begins early in childhood, leading to divergent life experiences for males and females that likely influence the lifespan development of the brain. Similarly, early postnatal factors such as exposure to sensory stimuli, environmental toxins, and feeding might also impact brain development, although less is known about whether this occurs in sex-specific ways. Investigating brain development over the neonatal period can provide an insight into how these early postnatal factors potentially influence sex differences in brain structure.

The neonatal period is typically defined as the first 4 weeks of life, and existing studies in the field typically involve infants with mean post-birth ages that extend beyond the neonatal period (e.g., 33 days post-birth in 13). As a result, an understanding of sex differences immediately after birth remains extremely limited. The majority of existing research has shown that, during early infancy, males have larger intracranial and total brain volumes than females [13–17], often even after accounting for birth weight. However, one study has reported no differences in intracranial or total brain volumes in 2–5 week-olds [18], contradicting these prior studies. Male infants are also reported to have larger total gray and white matter volumes, though these differences do not persist after accounting for the sex difference in intracranial volume [14, 15]. However, when controlling for total brain volume rather than intracranial volume, another study has reported that 1 month-old males had larger total white matter volumes, whilst females had larger total gray matter volumes [17]. These observed discrepancies emphasise the need for further research to clarify sex differences in the neonatal brain.

Research into brain regional sex differences is even more limited and inconsistent, complicating the identification of regions that show reliable sex differences during early development. When using region-of-interest volumetry, one study reported no regional sex differences in early infancy after controlling for intracranial volume [14]. However, when using voxel-based approaches such as tensor- and deformation-based morphometry, other studies have reported various regional sex differences even after controlling for brain size [13, 14]. For instance, male infants had increased gray matter volumes in the insula, middle temporal gyrus, fusiform gyrus, and hippocampus, whilst female infants had increased volumes in the dorsolateral prefrontal, motor, and visual cortices [14].

In summary, a limited number of studies have investigated sex differences in neonatal brain structure. This gap is surprising as the prenatal and neonatal periods are amongst the most rapid periods of brain development [19–21] and are likely critical windows for understanding sex differences in brain development. Moreover, given that brain development is highly dynamic during the first few weeks of life, existing findings from later stages of infancy cannot necessarily be extrapolated to the neonatal period. Neonatal research also provides a pivotal opportunity to understand the origins of sex differences in the brain and, specifically, the role of prenatal and early postnatal development in shaping these differences. To address this knowledge gap, we leveraged a sample of 514 newborns from the developing Human Connectome Project (dHCP) to assess sex differences in global and regional brain volumes. We further incorporated sex-by-age interactions in our analysis to investigate sex differences in early postnatal brain development and understand the potential role early postnatal factors play in shaping these sex differences.

## Methods

### Participants

Participants were recruited as part of the developing Human Connectome Project (dHCP) [22] which was ethically approved by the UK National Research Ethics Authority (14/LO/1169). The dHCP contains data from 783 newborn infants [22]. The exclusion criteria employed in this study included preterm births (< 37 weeks gestational age), multiple births, the presence of brain anomalies in the scan with likely analytical and clinical significance (determined by an expert perinatal neuroradiologist), a postnatal age > 28 days at the time of the scan, and pregnancy or neonatal clinical complications. The final sample used in this research consisted of 514 (236 birth-assigned females, 278 birth-assigned males) healthy, term-born, singleton infants scanned within the first 0–28 days of life (see Tables 1 and 2 for sample characteristics). Of these, 292 (56.8%) of infants were scanned within the first 7 days of birth (see Supplementary Figure S1 for distribution of infant postnatal age at the time of the MRI scan).

**Table 1.** Sample characteristics

	Mean (SD) All	Mean (SD) males	Mean (SD) females
Gestational age at birth (weeks)	40.15 (1.17)	40.11 (1.14)	40.21 (1.20)
Postnatal age at scan (days)	8.69 (8.20)	8.24 (7.85)	9.21 (8.58)
Postconceptional age at scan (weeks)	41.39 (1.62)	41.29 (1.57)	41.52 (1.67)
Birth weight (kg)	3.44 (0.48)	3.50 (0.46)	3.36 (0.49)
Head circumference (cm)	35.22 (1.64)	35.49 (1.55)	34.91 (1.69)
Maternal age (years)	33.64 (4.81)	33.62 (4.65)	33.67 (4.99)
Paternal age (years)	36.03 (6.15)	35.82 (6.31)	36.28 (5.96)

**Table 2.** Maternal ethnicity

		Full sample	Male	Female
Maternal ethnicity	White	320 (62.34%)	164 (58.99%)	156 (66.10%)
	Asian/Asian British	48 (9.33%)	23 (8.27%)	25 (10.59%)
	White and Asian	4 (0.78%)	0 (0%)	4 (1.69%)
	Black/Black British	70 (13.62%)	42 (15.10%)	28 (11.86%)
	White and Black African/Caribbean	6 (1.17%)	5 (1.80%)	1 (0.42%)
	Chinese	21 (4.09%)	14 (5.04%)	7 (2.97%)
	Any other mixed ethnic group	10 (1.95%)	7 (2.52%)	3 (1.27%)
	Any other ethnic group	27 (5.25%)	16 (5.75%)	11 (4.66%)
	Do not wish to answer	8 (1.56%)	7 (2.52%)	1 (0.42%)

### Data acquisition

Data collection took place at the Evelina Newborn Imaging Centre, Evelina London Children’s Hospital. Data was acquired on a 3-Tesla Philips Achieva system (Philips Medical Systems) using the dHCP neonatal brain imaging system, which included a neonatal 32 channel phased array head coil and a customised patient handling system (Rapid Biomedical GmbH, Rimpar, Germany, 23). Infants were scanned without sedation after being fed and swaddled in a vacuum-evacuated blanket. For auditory protection, infants were equipped with earplugs (President Putty, Coltene Whaledent, Mahwah, NJ, USA) and neonatal earmuffs (MiniMuffs, Natus Medical Inc., San Carlos, CA, USA). Heart rate, oxygen saturation, and temperature were monitored throughout the scan by a paediatrician or neonatal nurse [22], and scans were discontinued if these values fell in the abnormal range. These values fell within the normal range for all participants included in this analysis.

Anatomical data acquisition was conducted according to the specifications in the dHCP protocol [22]. The imaging parameters were optimised to maximise contrast-to-noise ratio using a Cramer Rao Lower bound approach (Lankford and Does, 2013). Nominal relaxation times were set at T1/T2: 1800/150ms for gray matter and at T1/T2: 2500/250 ms for white matter [24]. T2-weighted and T1-weighted inversion recovery Fast Spin Echo (FSE) images were obtained in sagittal and axial planes. In-plane resolution was set at 0.8 × 0.8 mm^2^ with a slice thickness of 1.6 mm with 0.8 mm overlap. T1-weighted sagittal images used a slice overlap of 0.74 mm. Other parameters were as follows - T2-weighted images: TR/TE = 12000/156 ms, SENSE factor 2.11 (axial) and 2.60 (sagittal); T1-weighted images: TR/TI/TE = 4795/1740/8.7 ms, SENSE factor 2.27 (axial) and 2.66 (sagittal). Additionally, 3D MPRAGE images were acquired using the following parameters: isotropic resolution = 0.8 mm, TR/TI/TE = 11/1400/4.6 ms, SENSE factor 1.2 RL (Right-Left). These acquisitions were optimised for volumetric analysis using a motion correction algorithm, and transverse and sagittal images were fused into a single 3D volume for high resolution and accurate volume estimation [25].

### Data preprocessing

The developing Human Connectome Project structural preprocessing pipeline was used for pre-processing the MRI data [26]. To summarise, the T2-weighted images were first motion-corrected, bias-corrected and brain-extracted using the Brain Extraction tool [27]. Next, a probabilistic tissue atlas was registered to the bias-corrected T2 image. Initial segmentation into different tissue types (i.e., cerebrospinal fluid, white matter, cortical gray matter, and subcortical gray matter) was performed using the Draw-EM algorithm [28]. Labelled atlases [29] were then registered to the subject’s images via a multi-channel registration process, using both GM probability maps from the initial segmentation and intensity T2-weighted images. The resulting segmentation consisted of 87 gray and white matter structures (see [28–30]).

### Statistical analysis

Statistical analysis were conducted on R (version 4.3.3, 2024-02-29), using the packages rstatix, tidyverse, effectsize, and ggplot2. Analysis of Covariance (ANCOVA) models were used to test for sex differences in brain volumes. Postconceptional age at the time of the MRI scan was used as a covariate in all models assessing for main effects of sex. Both absolute (without accounting for sex differences in size) and relative (accounting for sex differences in size) analyses were conducted. To account for sex differences in size, birth weight (global and regional analyses) and total brain volume (regional and total gray/white matter analyses) were included as covariates across separate models. The measure of total brain volume was derived by summing the volumes of all cortical and subcortical structures excluding the ventricles and cerebrospinal fluid. To facilitate cross-study comparability with studies that have used intracranial volume as a covariate, further regional analyses were conducted controlling for intracranial rather than total brain volume. The measure of total intracranial volume was derived by adding cerebrospinal fluid volume to total brain volume. Regional analyses focused primarily on gray matter volumes and were conducted on 47 cortical and subcortical gray matter regions [24]. The white matter volume of the corpus callosum, however, was also included in the analysis since sex differences in the corpus callosum are of key interest due to its critical role in inter-hemispheric connectivity [31, 65]. To investigate sex differences in neonatal brain development, we also conducted ANOVAs assessing for interactions between sex and postconceptional age at scan across all global and regional volumes. Analyses were corrected for multiple comparisons using the Benjamini-Hochberg FDR correction [32] with a significance threshold of 0.05. FDR corrections were run separately for global volumes (6 tests) and regional volumes (48 tests) for each analysis model. Effect sizes were estimated using partial eta squared [33], which provided a measure of the proportion of variance in brain volumes explained by sex after accounting for other covariates. 0.01 was considered a small effect, 0.06 a medium effect, and 0.14 a large effect [33]. Additionally, Welch’s two-sample t-tests were used to assess sex differences in continuous sample characteristics (e.g., postconceptional age at scan, postnatal age at birth, birth weight, etc.) and Pearson’s chi-squared tests were used to assess sex differences in categorical sample characteristics (e.g., ethnicity).

## Results

### Sample characteristics

Welch’s two-sample t-tests showed no significant differences between males and females in gestational age at birth (*p*_*FDR*_* =* 0.502*, d =* 0.08), postnatal age at scan (*p*_*FDR*_* =* 0.315*, d =* 0.12*)*, postconceptional age at scan (*p*_*FDR*_* =* 0.233*, d =* 0.15), maternal age (*p*_*FDR*_* =* 0.910*, d =* 0.01), or paternal age (*p*_*FDR*_* =* 0.502*, d =* 0.07). There was a significant difference in birth weight (*p*_*FDR*_* =* 0.004*, d =* 0.30) and head circumference (*p*_*FDR*_* =* < 0.001*, d =* 0.36), both of which were greater in males (see Table 1). Pearson’s chi-squared tests indicated no significant sex differences in maternal ethnicity (*p*_*FDR*_ ***=*** 0.308).

### Global analyses

**Fig. 1:**
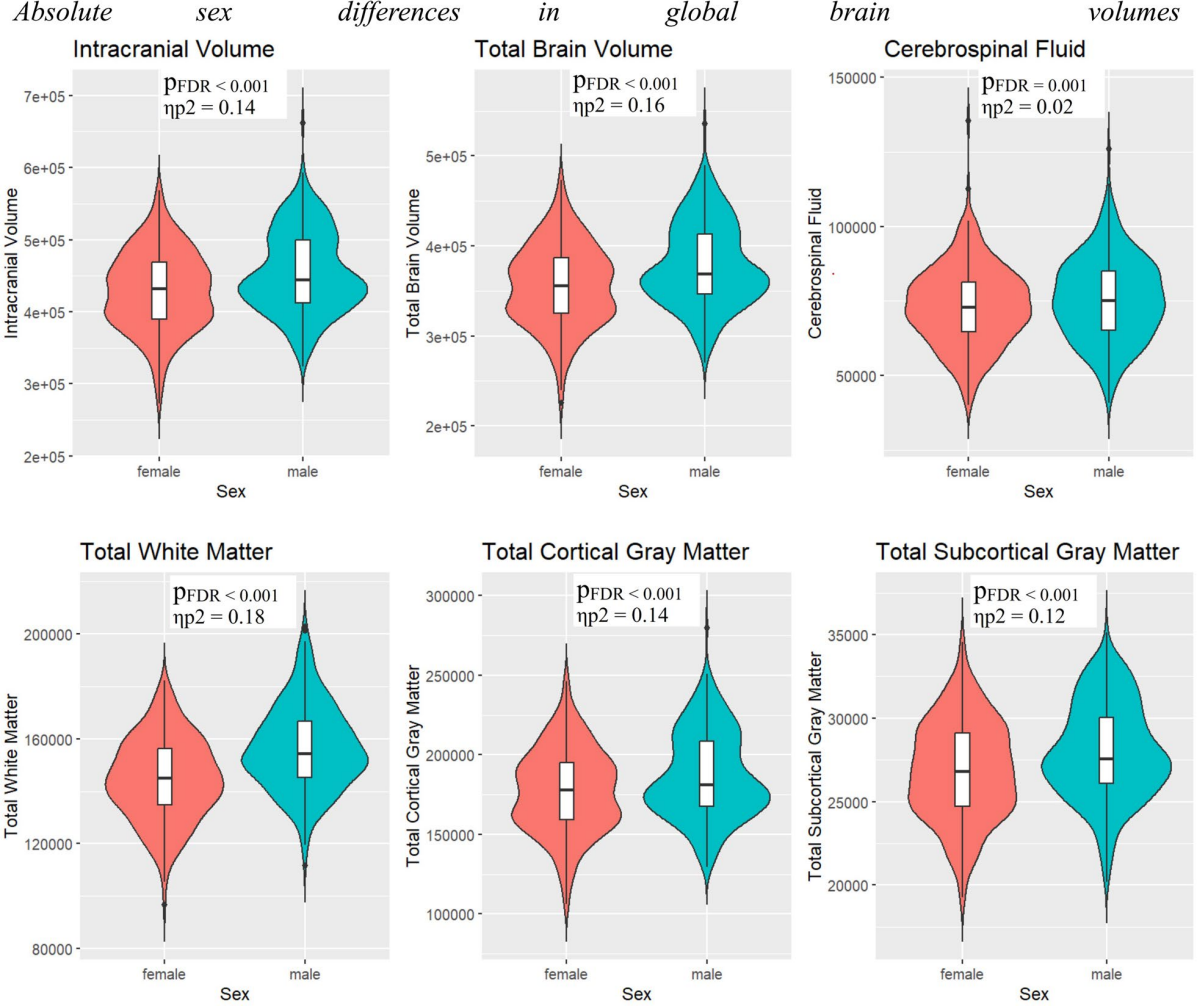
Absolute sex differences in global brain volumes All volumes are in mm^3^. *p*_*FDR =*_ FDR-corrected *p* value, η_p_^2^ = partial eta squared.

ANCOVA models were used to test for sex differences in global and regional brain volumes. After controlling for postconceptional age at scan and correcting for multiple comparisons, all global brain volumes (Fig. 1 and Supplementary Table S1) were larger in males than in females (all FDR-corrected *p *< 0.001). All these differences remained significant after controlling for birth weight (all FDR-corrected *p *< 0.001), except for the sex difference in cerebrospinal fluid (*p*_*FDR*_ = 0.134, η_p_^2^ = 0.02) (Table 3). After controlling for total brain volume in place of birth weight, males had larger total white matter volumes than females (*p*_*FDR*_ = 0.004, η_p_^**2**^ = 0.02), whereas females had larger cortical gray matter volumes than males (*p*_*FDR*_ = 0.023, η_p_^2^ = 0.01). There was no sex difference in total subcortical gray matter volumes (*p*_*FDR*_ = 0.249, η_p_^2^< 0.01) (Table 3).

**Table 3.** Sex differences in global brain volumes controlled for birth weight or total brain volume

	Controlled for birth weight			Controlled for total brain volume		
	F	*p*_*FDR*_	η_p_^2^	F	*p*_*FDR*_	η_p_^2^
Total intracranial volume	46.12	< 0.001 (M > F)	0.14	–	–	–
Total brain volume	59.29	< 0.001 (M > F)	0.18	–	–	–
Cerebrospinal fluid	2.26	0.134	0.01	–	–	–
Total cortical gray matter	46.77	< 0.001 (M > F)	0.14	5.21	0.023 (F > M)	0.01
Total white matter	69.23	< 0.001 (M > F)	0.20	8.17	0.004 (M > F)	0.02
Total subcortical gray matter	28.59	< 0.001 (M > F)	0.09	1.33	0.249	< 0.01

All analyses are additionally controlled for postconceptional age at scan. All *p*-values are FDR-corrected for multiple comparisons across the six analyses. All volumes are in mm^3^. F = F statistic, *p*_*FDR =*_ FDR-corrected *p* value, η_p_^2^ = partial eta squared.

### Regional analysis

**Fig. 2:**
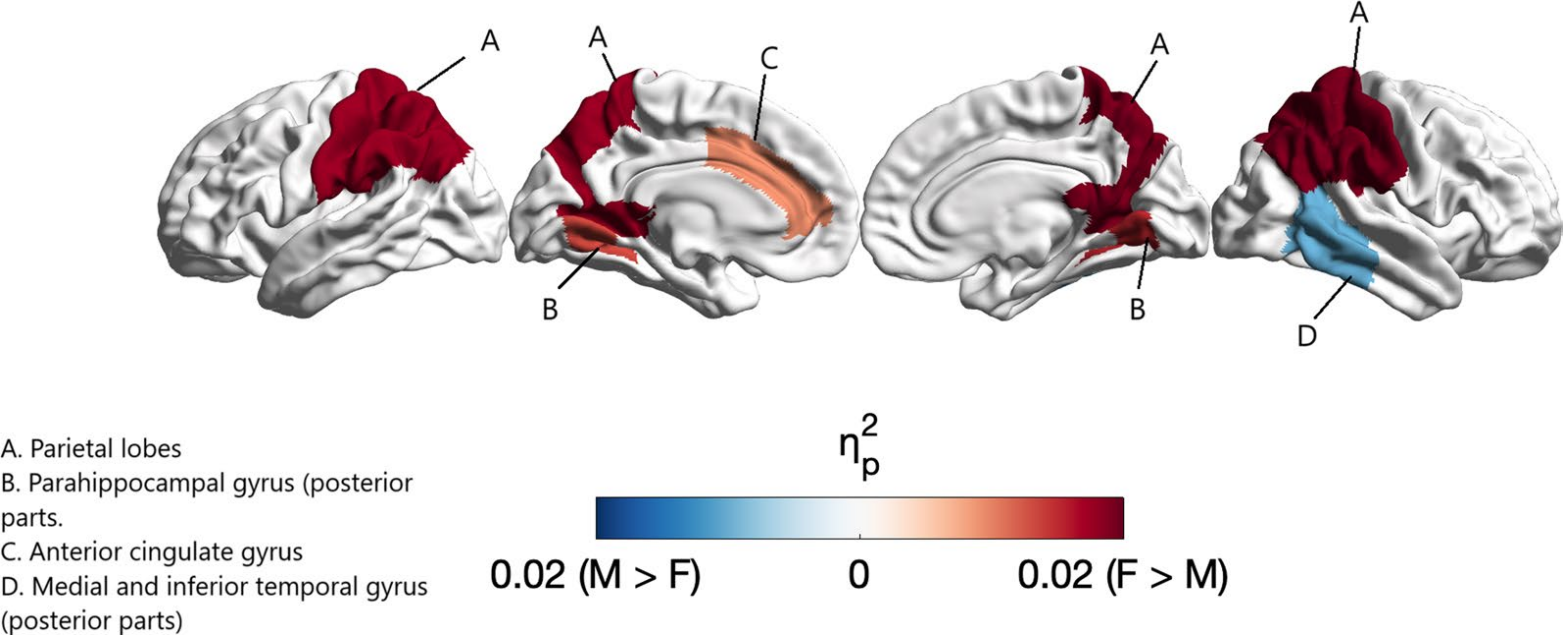
Regional sex differences by effect size after controlling for total brain volume. Figure 2 depicts partial eta-squared (ηp2 ) values of cortical regions showing significant sex differences projected on a 32k Conte69 midthickness

After controlling for postconceptional age at scan, all regional volumes were larger in males (all FDR-corrected *p*< 0.01). Full results of this analysis are reported in Supplementary Table S2. All of these regions remained significant after further controlling for birth weight (Supplementary Table S5). When controlling for total brain volume, female > male sex differences were observed in 7 regions, including the white matter volume of the corpus callosum (*p*_*FDR*_ = 0.026, η_p_^2^ = 0.01) and the gray matter volumes of the left (*p*_*FDR*_ = 0.015, η_p_^2^ = 0.01) and right (*p*_*FDR*_ = 0.010, η_p_^2^ = 0.02) parahippocampal gyri (posterior parts), left anterior cingulate gyrus (*p*_*FDR*_ = 0.042, η_p_^2^ = 0.01), left (*p*_*FDR*_ = 0.004, η_p_^2^ = 0.02) and right (*p*_*FDR*_ = 0.003, η_p_^2^ = 0.02) parietal lobes, and left caudate nucleus (*p*_*FDR*_ = 0.018, η_p_^2^ = 0.01) (Table 4). Male > female gray matter regions were observed in 2 regions, including the right medial and inferior temporal gyrus (posterior part) (*p*_*FDR*_ = 0.034, η_p_^2^ = 0.01) and right subthalamic nucleus (*p*_*FDR*_ = 0.043, η_p_^2^ = 0.01) (Table 5). These results are summarised in Tables 4 and 5 and visualised in Fig. 2. Full results are reported in Supplementary Table S3.

**Table 4.** Female > male regions after controlling for total brain volume

Region	Male mean (SE)	Female mean (SE)	F	*p*_*FDR*_	η_p_^2^
Right parahippocampal gyrus (posterior part)	815 (5.34)	838 (5.84)	7.81	0.010	0.02
Left parahippocampal gyrus (posterior part)	798 (5.78)	821 (6.32)	6.89	0.015	0.01
Left anterior cingulate gyrus	1314 (11.00)	1352 (12.10)	4.88	0.042	0.01
Right parietal lobe	18319 (50.10)	18563 (54.80)	9.95	0.003	0.02
Left parietal lobe	18454 (49.10)	18685 (53.70)	9.26	0.004	0.02
Left caudate nucleus	1879 (10.70)	1921 (11.70)	2.97	0.018	0.01
Corpus callosum	2909 (18.90)	2979 (20.70)	5.82	0.026	0.01

All *p-*values are FDR-corrected for multiple comparisons across 48 regions. Means values are estimated marginal (EM) means controlled for total brain volume and postconceptional age at scan (absolute means are reported in supplementary materials). All volumes are in mm^3^. F = F statistic, *p*_*FDR =*_ FDR-corrected *p* value, η_p_^2^ = partial eta squared.

**Table 5.** Male > female regions after controlling for total brain volume

Region	Male mean (SE)	Female mean (SE)	F	*p*_*FDR*_	η_p_^2^
Right medial and inferior temporal gyrus (posterior part)	3947 (17.80)	3883 (19.50)	5.30	0.034	0.01
Right subthalamic nucleus	220 (1.06)	216 (1.15)	4.83	0.043	0.01

All *p*-values are FDR-corrected for multiple comparisons across 48 regions. Means values are estimated marginal (EM) means controlled for total brain volume and postconceptional age at MRI scan. All volumes are in mm^3^. F = F statistic, *p*_*FDR =*_ FDR-corrected *p* value, η_p_^2^ = partial eta squared**.**

Full results for the model controlling for intracranial volume in place of total brain volume are reported in Supplementary Table S4 and Supplementary Figure S2. To summarise, female > male differences remained in the corpus callosum (*p*_*FDR*_ = 0.015, η_p_^2^ = 0.01) and left parahippocampal gyrus (posterior parts) (*p*_*FDR*_ = 0.043, η_p_^2^ = 0.01), and male > female differences remained in the right medial and inferior temporal gyri (posterior parts) (*p*_*FDR*_ = 0.002, η_p_^2^ = 0.01) and right subthalamic nucleus (*p*_*FDR*_ = 0.005, η_p_^2^ = 0.02). A number of additional male > female differences were observed in the bilateral insula (left: *p*_*FDR*_ = 0.003, η_p_^2^ = 0.02, right: *p*_*FDR*_ = 0.004, η_p_^2^ = 0.02), bilateral amygdala (left: *p*_*FDR*_ = 0.023, η_p_^2^ = 0.01, right: *p*_*FDR*_ = 0.043, η_p_^2^ = 0.02), left subthalamic nucleus (*p*_*FDR*_ = 0.020, η_p_^2^ = 0.01), bilateral superior temporal gyrus (middle part) (left: *p*_*FDR*_ = 0.024, η_p_^2^ = 0.01, right: *p*_*FDR*_ = 0.036, η_p_^2^ = 0.01), left anterior temporal lobe (lateral part) (*p*_*FDR*_ = 0.045, η_p_^2^ = 0.00), and right frontal lobe (*p*_*FDR*_ = 0.016, η_p_^2^ = 0.01).

### Sex-by-age interactions

**Fig. 3:**
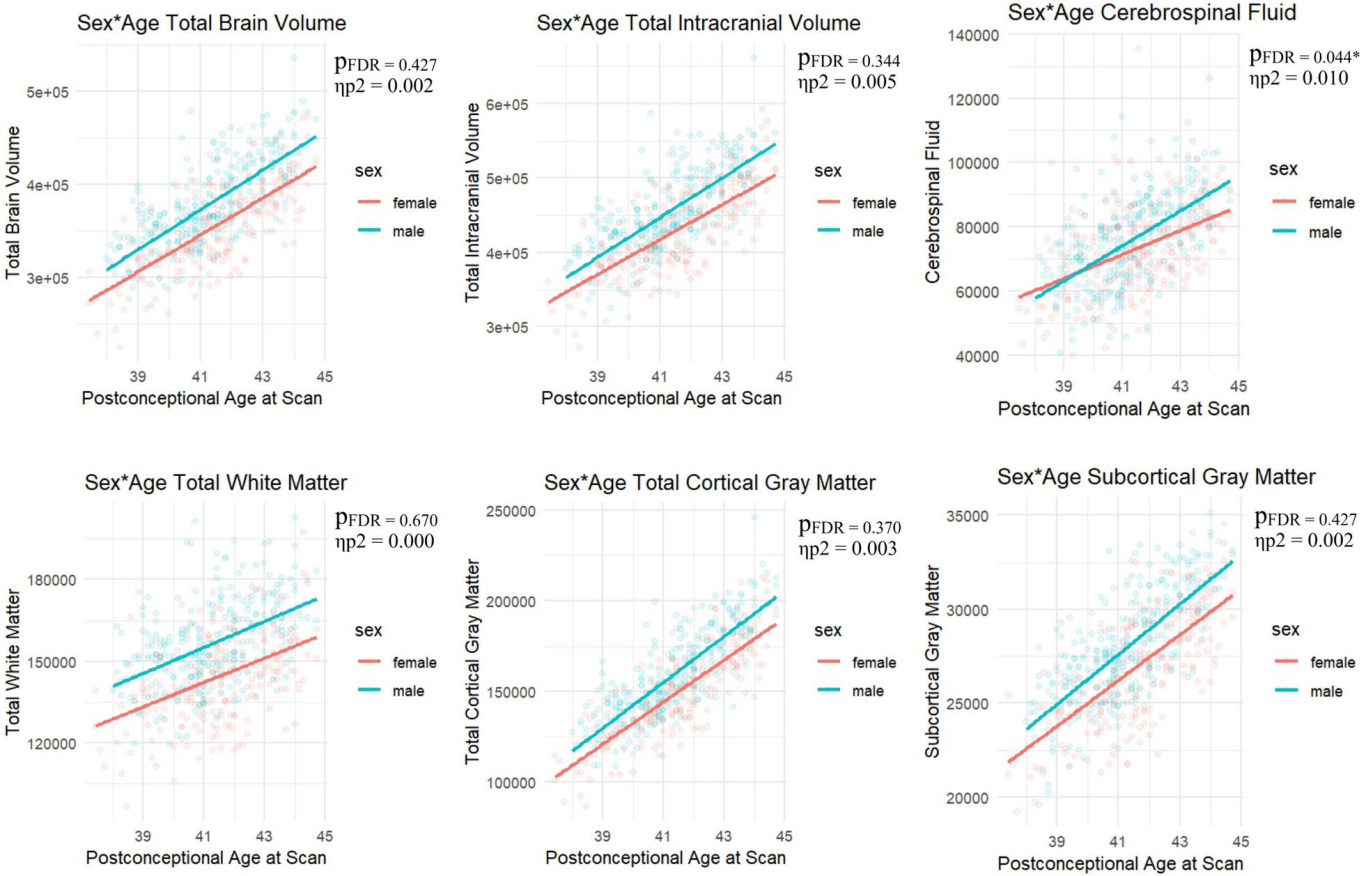
Global Sex-by-Age interactions. All p-values are FDR-corrected for multiple comparisons across the 6 analyses. All volumes are in mm^3^. *p*_*FDR =*_ FDR-corrected *p* value, η_p_^2^ = partial eta squared.

**Fig. 4:**
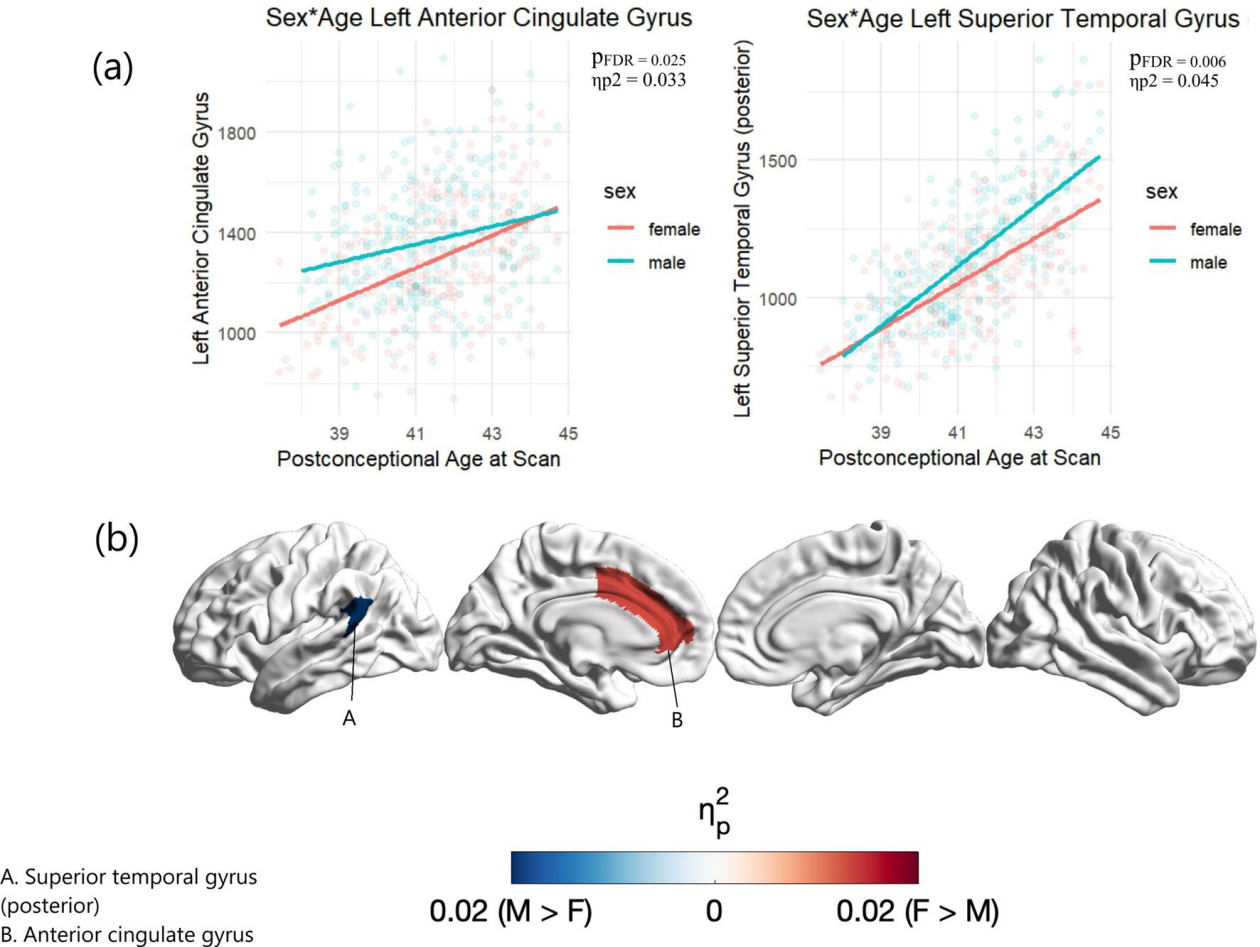
Regional Sex-by-Age Interactions

None of the global brain volumes showed significant sex-by-age interactions (see Fig. 3 and Supplementary Table S6) except for cerebrospinal fluid, where males showed increasing volumes with age compared to females (*p*_*FDR*_ = 0.040, η_p_^2^ = 0.010). Significant regional sex-by-age interactions were identified in the left superior temporal gyrus (posterior part) (*p*_*FDR*_ = 0.045, η_p_^2^= 0.05), where males showed increasing volumes with age compared to females, and left anterior cingulate gyrus (*p*_*FDR*_ = 0.025, η_p_^2^ = 0.03), where females showed increasing volumes with age compared to males (see Table 6 and Fig. 4). Full results are reported in Supplementary Table S6. A similar pattern of results was observed when including total brain volume or intracranial volume as a covariate for regional and total gray and white matter analyses (Supplementary Table S7 and S8).

**Table 6.** Significant Regional Sex-by-Age Interactions

Region	F	*p*_*FDR*_	η_p_^2^	Direction of interaction
Left anterior cingulate gyrus	5.75	0.025	0.03	Female > male
Left superior temporal gyrus (posterior part)	8.31	0.006	0.05	Male > female

All p-values are FDR-corrected for multiple comparisons across the 48 regions. F = F statistic, *p*_*FDR =*_ FDR-corrected *p* value, η_p_^2^ = partial eta squared**.**

Figure 4a *pFDR* = FDR-corrected *p *value, ηp2 = partial eta squared. Figure 4b depicts partial eta-squared (ηp2) values of regions showing significant sex-by-age interactions (*pFDR* < 0.05) projected on a 32 k Conte69 midthickness.

## Discussion

Despite being critical and dynamic stage for brain development, sex differences in the neonatal period (first 28 days of life) remain surprisingly underexplored. Studying this period can clarify how early on in development sex differences are present in the brain and how prenatal and early postnatal factors contribute to these differences. In this research, we identified a number of significant global and regional on-average sex differences in neonatal brain volumes. All absolute global brain volumes were significantly larger in males with large effect sizes, even after controlling for birth weight. After controlling for total brain volume, females showed significantly increased total cortical gray matter volumes while males showed increased total white matter volumes. After controlling for total brain volume, various significant regional female > male (e.g., corpus callosum, posterior parts of the bilateral parahippocampal gyri, parietal lobes, left caudate nucleus, left anterior cingulate gyrus) and male > female (e.g., posterior right medial and inferior temporal gyrus) differences were identified with small effect sizes. Few significant sex-by-age interactions were identified, except for in the left anterior cingulate gyrus (F > M) and left superior temporal gyrus—posterior parts (M > F). As discussed further below, these findings suggest that several sex differences observed later in life are already present at birth and remain relatively stable during the neonatal period.

### Sex differences in global volumes

First, we replicated the consistently reported finding that males have significantly larger total brain (by 6.16%) and intracranial (by 5.64%) volumes than females, even after controlling for birth weight. The presence and magnitude of these differences is largely consistent with prior research in early infancy [13–15]. These findings therefore confirm that sex differences in total brain volume are present from birth and are not fully accounted for by differences in body size. It is noteworthy that a meta-analysis [1] has previously reported 12% larger intracranial and 10.8% larger total brain volumes in males than females across the lifespan. Thus, although present at birth, these sex differences appear to increase in magnitude over the course of development. After controlling for total brain volume, females had significantly larger total cortical gray matter volumes whilst males had larger total white matter volumes. This finding is largely consistent with research in later life stages [34–40]. Collectively, these findings suggest that sex differences in global brain volumes are present from birth and are observed consistently throughout subsequent life stages.

### Sex differences in regional volumes

After controlling for total brain volume, females had significantly larger gray matter volumes in regions such as the bilateral parahippocampal gyri (posterior parts), left anterior cingulate gyrus, bilateral parietal lobes, and left caudate nucleus. Greater parietal lobe volumes in females have also been previously reported in early infancy [13, 18]. Moreover, adult females show higher gray to white matter ratios [41–43] and greater cortical thickness in the parietal lobe [44–47] than adult males. Interestingly, prior work has also suggested a negative association between adolescent circulating testosterone levels and parietal lobe volumes [48]. A previous meta-analysis has reported larger volumes in the posterior parts of the parahippocampal gyrus in females [1]. Similarly, numerous studies across the lifespan [48, 50–55], including research in young infants [16, 17], have also reported a larger caudate nucleus in females. The caudate nucleus, part of the basal ganglia, shows a high density of sex-steroid receptors [56, 57]. Moreover, the caudate nucleus has been implicated in a number of conditions that show sex differences in their prevalence, such as ADHD [58, 59], Tourrete’s syndrome [60], depression [61, 62], and autism [63] Finally, female newborns had relatively larger white matter volumes in the corpus callosum. An extensive body of previous research across various life stages supports the present findings [22, 64–68]. It has been suggested that a larger corpus callosum may explain the lower hemispheric asymmetry observed in females [69, 70]. Aspects of the corpus callosum, including its lateralisation and symmetry, also show associations with fetal testosterone levels [71]. Importantly, the corpus callosum has been implicated in conditions that show sex differences and manifest during early childhood [72, 73].

On the other hand, males showed significantly greater gray matter volumes in the subthalamic nucleus and the right medial and inferior temporal gyri (posterior parts) after controlling for total brain volume. Sex differences in the subthalamic nucleus have not been reported by prior research in later life, indicating that this sex difference might be unique to the neonatal stage. Regarding the medial and inferior temporal gyri, research in adolescents and adults corresponds with the present neonatal findings [28, 74]. However, another study in early infancy has reported that the posterior parts of the medial and inferior temporal gyri were larger in females. It is important to note that the sample in this prior study included pre-term and twin infants, who are known to show different brain phenotypes and developmental trajectories compared to term-born, singleton infants [75, 76]. These differences in sample characteristics, the use of a voxel-wise (tensor-based morphometry) rather than parcellation approach, and controlling for intracranial rather than total brain volume might explain the discrepancies with the present findings.

The decision to control for intracranial or total brain volume is an important one as the two approaches often yield different results, which perhaps stands as a leading source for inconsistency between existing studies in the field [2]. In this study, we present findings from both approaches to facilitate cross-study comparability. Sex differences that were consistent across the two analyses included significant female > male differences in the corpus callosum and left parahippocampal gyrus (posterior parts), and significant male > female differences in the right subthalamic nucleus and right medial and inferior temporal gyrus (posterior parts). However, controlling for intracranial volume also yielded a number of additional male > female differences in regions such as the bilateral amygdala, bilateral insula, and right frontal lobe—all of which have also been documented by prior research [1, 49]. This pattern aligns with prior studies wherein controlling for intracranial rather than total brain volume typically shows a greater number of male > female differences [77, 78]. The trend likely links to our finding that males continue to have larger total brain volumes even after controlling for intracranial volume, which might explain why male > female differences attenuate when controlling for total brain volume itself.

### Sex-by-age interactions

Significant sex-by-age interactions were identified in the left anterior cingulate gyrus, where females showed increasing volumes with age, and left superior temporal gyrus (posterior parts), where males showed increasing volumes with age. A larger anterior cingulate gyrus in females has previously been reported in early infancy [14, 17] and in a large sample of 2328 adults [49]. The findings reported here indicate that sex differences in the anterior cingulate gyrus amplify during early postnatal development. Most other global and regional volumes that showed main effects of sex did not show significant sex-by-age interactions, indicating that sex differences in these regions remain relatively stable during early postnatal development.

### Sex differences across development

More broadly, three patterns appear to emerge by synthesising the findings of the present neonatal research with those from later life stages: (a) some sex differences observed throughout the lifespan appear to be present from birth; (b) some sex differences are absent at birth but present in later development; and (c) some sex differences are present at birth but absent in later development. Pattern (a) appears to be most prevalent in our findings, having been observed in all global brain volumes as well as various regional volumes (e.g., caudate nucleus, anterior cingulate cortex, corpus callosum, etc.). It has previously been proposed that sex differences can be categorised as either “persistent”, such that they are established early in development and persist throughout the lifespan, or “transient”, such that they are temporary to a specific developmental period [79]. Under this framework, the findings identified in pattern (a) can be classified as persistent sex differences, although these differences might still be dynamic over development. For instance, the sex difference in brain size is persistent in the sense that it is present from birth, but dynamic in the sense that it increases in magnitude over the course of development.

Regarding pattern (b), sex differences typically observed in adults that we did not observe in this neonatal sample are seen in regions such as the hippocampus and fusiform gyrus [1, 49]. These sex differences might manifest as a result of both environmental influences as well as biological factors that unfold over development. Findings falling under pattern (c) include the subthalamic nucleus and can be understood as transient sex differences that might emerge as a result of short-term effects of prenatal processes. Although these differences are no longer observed during later development, they might play some initial role in instigating sex-specific developmental trajectories. Going forward, it will be important to verify these patterns via further longitudinal research on sex differences over the lifespan. Recent work on brain structural changes throughout the lifespan [19] and subcortical development during early childhood [16] set examples for future research to build upon.

### Strengths and limitations

There are important considerations that need to be taken into account when interpreting the findings of this study. First, the sample is not longitudinal, limiting the conclusions that can be drawn from analyses assessing sex-by-age interactions. Second, whilst it is reasonable to speculate that these sex differences may be influenced by prenatal factors (such as fetal testosterone), it is important to note that our findings do not establish any causal relationships between the two. Third, there might be a delayed effect of some prenatal biological processes, with their outcomes manifesting only gradually over development [7, 80]. This suggests that neonatal research might capture only those effects that are immediately observable, potentially missing later-emerging effects. Fourth, sex differences in brain structure are not necessarily synonymous with sex differences in brain function or behaviour [55, 81]. Further research directly examining these links will be essential to understanding whether the present findings have any implications for sex differences in behaviour and cognition. Fifth, given that definitions of regions can differ by atlas, cross-study compatibility of regional differences can be compromised [82]. Sixth, social determinants such as family income and maternal education levels have previously been shown to be associated with neonatal brain volumes (83), though these measures were not available in this dataset and could not properly be taken into consideration in this analysis. Finally, the present research examines only one of the many ways the brain can differ between males and females. Further research employing other neuroanatomical, diffusion-weighted, and functional measures will be critical to achieving a comprehensive insight into sex differences in the neonatal brain.

Strengths of the present research include the relatively large sample size. Importantly, the majority of infants were scanned within the first few days of birth, allowing us to capture the early neonatal period prior to extensive postnatal environmental influences. Moreover, the dHCP structural pre-processing pipeline [26] used in this research is optimised for the neonatal brain and overcomes several challenges typically encountered in neonatal brain imaging (e.g., partial volume effects, low tissue contrast, motion artefacts, etc.). The pipeline’s output also shows high correspondence with manual assessments of tissue boundaries.

### Perspectives and significance

It is possible that the early-emerging sex differences identified in this research influence neurobiological development from the very beginning of life, potentially explaining the sex differences observed in early-emerging neuropsychiatric and neurodevelopmental conditions. At present, the mechanisms linking sex differences in brain structure to these brain-based conditions remain poorly understood. Going forward, understanding this link should be an important research focus. The prenatal period might be a particularly important stage to study such links given that it appears to be a critical window for sex differences to manifest in the brain. Furthering this line of research can also ultimately contribute towards tailoring early diagnostic and support strategies based on sex.

## Conclusion

In conclusion, sex differences are well-evidenced across later development, but remain significantly underexplored during the neonatal period. Our findings suggest that sex differences in brain structure are present from the earliest stage of postnatal life and show an overlap with the sex differences observed in future stages of development. We report comparatively fewer sex-by-age interactions, indicating that several of these sex differences are established during the prenatal period and thereafter remain relatively stable during the neonatal period. The early emergence of these differences supports the hypothesis that prenatal factors play a pivotal role in initiating sex differences in the brain.

## Supplementary Information

Supplementary Material 1.

## Data Availability

The datasets generated and/or analysed during the current study are available via the developing Human Connectome Project, https://www.developingconnectome.org/data-release/
