# Supplementary material for: Sex Differences in Human Brain Structure at Birth
Source: Biol Sex Differ. 2024 Oct 17;15:81. doi: 10.1186/s13293-024-00657-5 (PMC11488075; doi:10.1186/s13293-024-00657-5)
Supplement: Supplementary file 1 — Supplementary Material 1. [file 13293_2024_657_MOESM1_ESM.pdf]

Supplementary Materials: Sex Differences in Human Brain Structure at Birth

Contents

Table S1. Absolute Sex Differences in Global Brain Volumes…………………………….…...….2

Table S2. Absolute Sex Differences in Regional Volumes……………………………………..….2-7

Table S3. Sex differences in regional volumes controlling for total brain volume..........................…7-12

Table S4. Sex Differences in regional volumes after controlling for intracranial volume…….……..12-16

Table S5. Sex Differences in regional volumes after controlling for birth weight…………….…..17-21

Table S6. Sex-by-age interactions……………………………………………………………..22-27

Table S7. Regional sex-by-age interactions controlling for total brain volume………………………27-32

Table S8. Regional sex-by-age interactions controlling for intracranial volume …………………….33-37

Figure S1. Distribution of Postnatal Age at Scan……………………………………………….….37

Figure S2. Regional sex differences by effect size after controlling for intracranial volume…………...38

Table S1.

Absolute sex differences in global brain volumes (mm³)

	**Male Mean (SD)**	**Female Mean (SD)**	**% difference**	**F**	p_{FDR}	η_p^2
Total intracranial volume	454471 (56273)	429533 (54615)	5.64%	84.76	<0.001	0.14
Total brain volume	378827 (45948)	356189 (44867)	6.16%	99.86	<0.001	0.16
Cerebrospinal fluid	75645 (13978)	73344 (13521)	3.08%	10.83	0.001	0.02
Total cortical gray matter	158723 (24474)	150295 (23648)	5.45%	81.67	<0.001	0.14
Total white matter	156497 (16062)	144564 (15642)	7.93%	109.55	<0.001	0.18
Total subcortical gray matter	27986 (2866)	26855 (2944)	4.12%	62.242	<0.001	0.12

All analyses are controlled for postconceptional age at scan. All *p*-values are FDR-corrected for multiple comparisons across the six analyses. All volumes are in mm³. **F** = F statistic, p_{FDR} = FDR-corrected *p* value, η_p^2 = partial eta squared.

Table S2. *Absolute Sex Differences in Regional Volumes*

**Region**	**Male Mean (SD)**	**Female Mean (SD)**	**F**	p_{FDR}	η_p^2
Hippocampus left	754.61 (108.54)	710.08 (96.14)	43.14	<0.001	0.048
Hippocampus right	843.27 (116.79)	794.69 (100.65)	47.16	<0.001	0.052

Amygdala left	493.06 (57.28)	464.99 (53.31)	57.96	*<0.001*	0.066
Amygdala right	560.85 (69.89)	528.73 (65.32)	53.87	<0.001	0.059
Anterior temporal lobe medial part left	950.06 (170.31)	892.01 (155.54)	37.71	<0.001	0.035
Anterior temporal lobe medial part right	987.10 (179.93)	931.49 (168.79)	36.53535	<0.001	0.030
Anterior temporal lobe lateral part left	1018.61 (195.79)	949.41 (194.67)	46.89	<0.001	0.040
Anterior temporal lobe lateral part right	1038.42 (188.05)	992.35 (197.97)	27.64	<0.001	0.021
Parahippocampal gyrus left (anterior part)	1152.14 (151.43)	1092.46 (140.68)	32.00	<0.001	0.041
Parahippocampal gyrus right (anterior part)	1035.84 (154.27)	992.35 (139.68)	21.89	<0.001	0.023
Superior temporal gyrus left (middle part)	2995.34 (485.12)	2818.21 (499.37)	62.24	<0.001	0.044
Superior temporal gyrus right (middle part)	3060.42 (487.87)	2882.87 (498.09)	60.88	<0.001	0.044

Medial and inferior temporal gyri left (anterior part)	2513.80 (490.85)	2364.28 (481.85)	42.91	<0.001	0.031
Medial and inferior temporal gyri right (anterior part)	2717.62 (536.70)	2582.42 (505.43)	38.76	<0.001	0.024
Lateral occipitotemporal gyrus left (anterior part)	738.82 (136.94)	703.06 (147.25)	21.75	<0.001	0.020
Lateral occipitotemporal gyrus right (anterior part)	782.66 (161.29)	742.26 (161.44)	23.81	<0.001	0.021
Cerebellum left	13274.96 (2187.81)	12914.43 (2163.71)	35.08	<0.001	0.012
Cerebellum right	12983.41 (2142.42)	12573.07 (2056.700)	41.06	<0.001	0.016
Brainstem	6711.41 (631.78)	6489.43 (631.78)	47.65	<0.001	0.038
Insula right	1768.60 (274.71)	1649.47 (258.102)	76.54	<0.001	0.058
Insula left	1956.75 (291.77)	1822.12 (261.383)	78.74	<0.001	0.067
Occipital lobe right	12211.90 (2041.58)	11665.80 (1940.91)	40.74	<0.001	0.027
Occipital lobe left	12332.42 (2036.91)	11787.81 (2015.79)	43.93	<0.001	0.027

Parahippocampal gyri right (posterior part)	837.90 (144.29)	811.61 (132.87)	15.76	<0.001	0.012
Parahippocampal gyri left (posterior part)	816.78 (127.02)	798.84 (130.97)	8.41	0.004	0.007
Latetal occipitotemporal gyus right (posterior part)	1027.06 (209.30)	988.73 (207.87)	19.02	<0.001	0.013
Lateral occipitotemporal gyrus left (posterior part)	978.36 (201.88)	937.59 (194.25)	16.69	<0.001	0.014
Medial and inferior temporal gyri right (posterior part)	4058.45 (713.17)	3751.90 (696.98)	88.78	<0.001	0.061
Medial and inferior temporal gyri left (posterior part)	4159.01 (753.13)	3864.00 (711.37)	76.32	<0.001	0.052
Superior temporal gyrus right (posterior part)	1295.30 (256.28)	1237.55 (233.245)	25.52	<0.001	0.019
Superior temporal gyrus left (posterior part)	1144.14 (239.22)	1094.56 (211.38)	23.94	<0.001	0.017
Cingulate gyrus right (anterior part)	1544.77 (252.26)	1433.81 (247.70)	44.87	<0.001	0.051
Cingulate gyrus left (anterior part)	1364.15 (234.38)	1293.74 (237.47)	17.41	<0.001	0.023
Cingulate gyrus right (posterior part)	1787.37 (309.72)	1652.96 (295.31)	52.99	<0.001	0.053

Cingulate gyrus left (posterior part)	1748.06 (301.37)	1643.19 (299.28)	40.26	<0.001	0.036
Frontal lobe right	26574.61 (4274.314)	24900.72 (4063.05)	90.83	<0.001	0.053
Frontal lobe left	26410.15 (4264.03)	24880.23 (4104.48)	80.80	<0.001	0.044
Parietal lobe right	18784.53 (3027.97)	18014.77 (2946.75)	53.25	<0.001	0.025
Parietal lobe left	18931.88 (3071.98)	18122.12 (2947.58)	55.85	<0.001	0.027
Caudate nucleus right	1989.20 (261.90)	1902.69 (265.61)	27.21	<0.001	0.028
Caudate nucleus left	1933.80 (256.78)	1856.97 (275.68)	22.02	<0.001	0.022
Thalamus right	4245.96 (442.83)	4102.72 (422.09)	46.35	<0.001	0.034
Thalamus left	4163.66 (433.66)	4012.22 (423.85)	53.84	<0.001	0.039
Subthalamic nucleus right	224.42 (26.22)	210.93 (26.55)	66.62	<0.001	0.072
Subthalamic nucleus left	187.45 (20.52)	176.62 (23.76)	48.81	<0.001	0.065
Lentiform nucleus right	3223.46 (437.95)	3074.19 (435.16)	52.17	<0.001	0.037
Lentiform nucleus left	3423.82 (421.25)	3256.58 (416.04)	60.40	<0.001	0.048

Corpus Callosum	2994.48 (477.23)	2878.41 (459.07)	19.29	<0.001	0.018

Note. All analyses are controlled for postconceptional age at scan. All *p*-values are FDR-corrected for multiple comparisons.

Table S3. *Sex Differences in regional volumes after controlling for total brain volume*

**Region**	**Male EM Mean (SE)**	**Female EM Mean (SE)**	**F**	p_{FDR}	η_p^2
Hippocampus left	737 (4.52)	731 (4.94)	0.54	0.504	0.001
Hippocampus right	824 (4.66)	817 (5.09)	0.82	0.418	0.002
Amygdala left	483 (2.36)	477 (2.58)	2.91	0.119	0.006
Amygdala right	549 (2.89)	542 (3.16)	2.41	0.163	0.005
Anterior temporal lobe medial part left	927 (7.00)	919 (7.66)	0.67	0.467	0.001
Anterior temporal lobe medial part right	959 (6.39)	965 (6.99)	0.32	0.604	0.001
Anterior temporal lobe lateral part left	978 (8.26)	994 (7.55)	1.88	0.216	0.004
Anterior temporal lobe lateral part right	1013 (7.09)	1022 (7.75)	0.58	0.493	0.001

Parahippocampal gyrus left (anterior part)	1124 (6.95)	1125 (7.60)	0.01	0.493	0.000
Parahippocampal gyrus right (anterior part)	1008 (6.40)	1025 (7.00)	3.05	0.113	0.006
Superior temporal gyrus left (middle part)	2932 (15.60)	2893 (17.10)	2.60	0.145	0.005
Superior temporal gyrus right (middle part)	2991 (15.2)	2964 (16.60)	1.35	0.298	0.003
Medial and inferior temporal gyri left (anterior part)	2438 (15.20)	2454 (16.70)	0.47	0.530	0.001
Medial and inferior temporal gyri right (anterior part)	2641 (15.00)	2673 (16.40)	1.87	0.216	0.004
Lateral occipitotemporal gyrus left (anterior part)	718 (5.98)	728 (6.54)	1.21	0.322	0.002
Lateral occipitotemporal gyrus right (anterior part)	760 (6.56)	769 (7.18)	0.78	0.432	0.002

Cerebellum left	13061 (54.10)	13166 (59.20)	1.55	0.265	0.003
Cerebellum right	12782 (54.70)	12811 (59.80)	0.12	0.754	0.000
Brainstem	6593 (18.40)	6629 (20.10)	1.55	0.263	0.003
Insula right	1726 (8.29)	1699 (9.06)	4.35	0.056	0.008
Insula left	1908 (8.79)	1880 (9.61)	4.32	0.056	0.008
Occipital lobe right	11891 (54.30)	12043 (59.40)	3.27	0.102	0.006
Occipital lobe left	12014 (50.30)	12163 (55.00)	3.65	0.081	0.007
Parahippocampal gyri right (posterior part)	815 (5.34)	838 (5.84)	7.81	0.010	0.020
Parahippocampal gyri left (posterior part)	798 (5.78)	821 (6.32)	6.89	0.015	0.015
Lateral occipitotemporal gyrus right (posterior part)	1004 (8.12)	1016 (8.88)	1.07	0.350	0.002

Lateral occipitotemporal gyrus left (posterior part)	9.53 (8.65)	967 (9.46)	1.10	0.347	0.002
Medial and inferior temporal gyri right (posterior part)	3947 (17.80)	3883 (19.50)	5.30	0.034	0.010
Medial and inferior temporal gyri left (posterior part)	4044 (19.40)	4000 (21.20)	2.20	0.183	0.004
Superior temporal gyrus right (posterior part)	1262 (9.21)	1277 (10.07)	1.06	0.350	0.002
Superior temporal gyrus left (posterior part)	1118 (8.75)	1126 (9.56)	0.38	0.575	0.001
Cingulate gyrus right (anterior part)	1493 (10.00)	1494 (11.00)	0.006	0.948	0.000
Cingulate gyrus left (anterior part)	1314 (11.00)	1352 (12.10)	4.88	0.043	0.009
Cingulate gyrus right (posterior part)	1722 (10.00)	1730 (10.90)	0.27	0.636	0.001
Cingulate gyrus left (posterior part)	1688 (9.65)	1714 (10.55)	2.87	0.123	0.005
Frontal lobe right	25836 (60.90)	25771 (66.59)	0.48	0.530	0.001

Frontal lobe left	25679 (59.33)	25742 (64.88)	0.47	0.530	0.001
Parietal lobe right	18319 (50.10)	18563 (54.80)	9.95	0.003	0.020
Parietal lobe left	18454 (49.10)	18685 (53.70)	9.26	0.004	0.020
Caudate nucleus right	1936 (10.70)	1965 (11.70)	2.97	0.118	0.006
Caudate nucleus left	1879 (10.70)	1921 (11.70)	6.58	0.018	0.012
Thalamus right	4167 (12.10)	4195 (13.30)	2.18	0.184	0.004
Thalamus left	4087 (11.60)	4102 (12.70)	0.650	0.465	0.001
Subthalamic nucleus right	220 (1.06)	216 (1.15)	4.83	0.043	0.009
Subthalamic nucleus left	184 (1.05)	181 (1.15)	3.10	0.111	0.006
Lentiform nucleus right	3159 (13.90)	3159 (13.90)	0.19	0.695	0.009
Lentiform nucleus left	3358 (13.90)	3334 (15.20)	1.33	0.299	0.003

Corpus Callosum	2909 (18.90)	2979 (20.70)	5.82	0.026	0.011

Note. All analyses are controlled for postconceptional age at scan and total brain volume. All *p* values are FDR-corrected for multiple comparisons. Means values are estimated marginalised means corrected for total brain volume and postconceptional age at birth.

Table S4. *Sex Differences in regional volumes after controlling for intracranial volume*

**Region**	**Male EM Mean (SE)**	**Female EM Mean (SE)**	**F**	p_{FDR}	η_p^2
Hippocampus left	739 (4.56)	729 (4.98)	2.09	0.198	0.004
Hippocampus right	826 (4.71)	814 (5.15)	2.74	0.137	0.005
Amygdala left	484 (2.61)	475 (2.61)	5.99	0.023	0.010
Amygdala right	551 (2.89)	541 (3.16)	4.81	0.043	0.009
Anterior temporal lobe medial part left	931 (7.12)	914 (7.78)	2.52	0.156	0.001
Anterior temporal lobe medial part right	963 (6.46)	960 (7.06)	0.05	0.870	0.001
Anterior temporal lobe lateral part left	999 (7.70)	973 (8.41)	4.710	0.045	0.004
Anterior temporal lobe lateral part right	1017 (7.17)	1017 (7.84)	0.00	0.988	0.001

Parahippocampal gyrus left (anterior part)	1128 (7.00)	1121 (7.65)	0.33	0.638	0.000
Parahippocampal gyrus right (anterior part)	1012 (6.51)	1021 (7.12)	0.81	0.438	0.002
Superior temporal gyrus left (middle part)	2941 (15.86)	2882 (17.32)	5.94	0.024	0.010
Superior temporal gyrus right (middle part)	3005 (15.92)	2949 (17.39)	5.18	0.036	0.010
Medial and inferior temporal gyri left (anterior part)	2451 (16.05)	2438 (17.54)	0.30	0.654	0.001
Medial and inferior temporal gyri right (anterior part)	2653 (15.55)	2659 (16.98)	0.07	0.844	0.000
Lateral occipitotemporal gyrus left (anterior part)	722 (6.14)	723 (6.71)	0.03	0.902	0.000
Lateral occipitotemporal gyrus right (anterior part)	764.38 (6.75)	763.79 (7.37)	<0.01	0.961	0.000

Cerebellum left	13092 (54.38)	13131 (59.40)	0.22	0.702	0.000
Cerebellum right	12812 (55.03)	12776 (60.12)	0.18	0.721	0.000
Brainstem	6608 (18.96)	6611 (20.71)	0.01	0.942	0.000
Insula right	1732 (8.50)	1692 (9.29)	9.25	0.004	0.020
Insula left	1916 (9.23)	1871 (10.08)	10.13	0.003	0.020
Occipital lobe right	11930 (54.94)	11999 (60.02)	0.666	0.482	0.001
Occipital lobe left	12051 (50.76)	12120 (55.45)	0.78	0.447	0.002
Parahippocampal gyri right (posterior part)	818 (5.39)	835 (5.88)	4.21	0.059	0.008
Parahippocampal gyri left (posterior part)	800 (5.75)	819 (6.28)	4.83	0.043	0.009
Lateral occipitotemporal gyrus right (posterior part)	1006 (8.06)	1014 (8.80)	0.38	0.612	0.001
Lateral occipitotemporal gyrus left (posterior part)	956 (8.58)	964 (9.37)	0.44	0.581	0.001

Medial and inferior temporal gyri right (posterior part)	3960 (17.94)	3868 (19.60)	10.94	0.002	0.010
Medial and inferior temporal gyri left (posterior part)	4060 (20.08)	3980 (21.93)	6.66	0.017	0.010
Superior temporal gyrus right (posterior part)	1267 (9.37)	1270 (10.24)	0.04	0.878	0.000
Superior temporal gyrus left (posterior part)	1122 (8.87)	1120 (9.69)	0.01	0.929	0.000
Cingulate gyrus right (anterior part)	1502 (10.60)	1484 (11.58)	1.29	0.315	0.003
Cingulate gyrus left (anterior part)	1323 (11.49)	1342 (12.55)	1.30	0.315	0.002
Cingulate gyrus right (posterior part)	1719 (11.58)	1731 (10.60)	0.61	0.503	0.001
Cingulate gyrus left (posterior part)	1731 (10.60)	1704 (10.97)	0.24	0.692	0.000
Frontal lobe right	25928 (66.82)	25662 (72.99)	6.70	0.016	0.010

Frontal lobe left	25768 (64.52)	25637 (70.48)	1.76	0.240	0.003
Parietal lobe right	18386 (55.12)	18484 (60.22)	1.33	0.314	0.003
Parietal lobe left	18517 (52.91)	18611 (57.80)	1.32	0.315	0.003
Caudate nucleus right	1946 (11.34)	1954 (12.39)	0.18	0.721	0.009
Caudate nucleus left	1889 (11.35)	1909 (12.40)	1.29	0.315	0.000
Thalamus right	4179 (12.77)	4182 (13.96)	0.02	0.906	0.003
Thalamus left	4098 (12.15)	4090 (13.28)	0.20	0.709	0.000
Subthalamic nucleus right	221 (1.08)	215 (1.18)	9.08	0.005	0.020
Subthalamic nucleus left	184 (1.07)	180 (1.16)	6.29	0.020	0.010
Lentiform nucleus right	3168 (14.10)	3140 (15.40)	1.72	0.241	0.003
Lentiform nucleus left	3368 (14.25)	3322 (15.57)	4.39	0.054	0.009
Corpus Callosum	2909 (17.59)	2980 (19.21)	6.90	0.015	0.010

All analyses are controlled for postconceptional age and intracranial volume. All *p* values are FDR-corrected for multiple comparisons. Means values are estimated marginalised means corrected for intracranial volume and postconceptional age at birth.

Table S5. Sex Differences in regional volumes after controlling for birth weight

**Region**	**Male Mean (SD)**	**Female Mean (SD)**	**F**	p_{FDR}	η_p^2
Hippocampus left	756.41 (5.39)	707.93 (5.85)	36.47	<0.001	0.067
Hippocampus right	844.80 (5.63)	792.62 (6.10)	38.80	<0.001	0.071
Amygdala left	493.72 (2.86)	464.19 (3.10)	48.02	<0.001	0.086
Amygdala right	561.87 (3.45)	527.48 (3.75)	44.77	<0.001	0.081
Anterior temporal lobe medial part left	955.49 (8.02)	884.68 (8.70)	35.13	<0.001	0.065
Anterior temporal lobe medial part right	992.06 (8.11)	924.96 (8.80)	30.84	<0.001	0.057
Anterior temporal lobe lateral part left	1024.39 (8.77)	942.07 (9.52)	39.71	<0.001	0.072
Anterior temporal lobe lateral part right	1044.00 (8.44)	985.64 (9.15)	21.57	<0.001	0.041
Parahippocampal gyrus left (anterior part)	1153.88 (8.19)	1089.79 (8.89)	27.58	<0.001	0.051

Parahippocampal gyrus right (anterior part)	1038.94 (7.86)	988.43 (8.53)	18.62	<0.001	0.035
Superior temporal gyrus left (middle part)	3012.47 (19.86)	2796.88 (21.54)	53.16	<0.001	0.095
Superior temporal gyrus right (middle part)	3077.52 (20.10)	2860.61 (21.81)	52.53	<0.001	0.094
Medial and inferior temporal gyri left (anterior part)	2528.04 (20.69)	2346.06 (22.44)	34.90	<0.001	0.064
Medial and inferior temporal gyri right (anterior part)	2729.20 (20.58)	2566.57 (22.33)	28.17	<0.001	0.052
Lateral occipitotemporal gyrus left (anterior part)	742.43 (6.98)	698.79 (7.58)	17.63	<0.001	0.033
Lateral occipitotemporal gyrus right (anterior part)	786.97 (7.60)	738.09 (8.25)	18.65	<0.001	0.035
Cerebellum left	13354.34 (71.12)	12812.51 (77.17)	26.17	<0.001	0.049
Cerebellum right	13059.25 (69.85)	12476.08 (75.79)	31.43	<0.001	0.058
Brainstem	6715.16 (26.80)	6483.79 (29.08)	33.60	<0.001	0.062

Insula right	1775.23 (11.30)	1639.28 (12.26)	65.33	<0.001	0.114
Insula left	1962.63 (12.31)	1813.34 (13.35)	66.37	<0.001	0.115
Occipital lobe right	12264.66 (80.31)	11594.00 (87.14)	31.45	<0.001	0.058
Occipital lobe left	12383.78 (77.69)	11719.21 (84.30)	32.99	<0.001	0.061
Parahippocampal gyri right (posterior part)	840.67 (6.60)	808.27 (7.16)	10.87	0.001	0.021
Parahippocampal gyri rleft (posterior part)	819.19 (6.57)	795.73 (7.13)	5.74	0.017	0.011
Latetal occipitotemporal gyus right (posterior part)	1032.95 (9.21)	980.83 (9.99)	14.45	<0.001	0.028
Lateral occipitotemporal gyrus left (posterior part)	982.68 (9.64)	931.97 (10.46)	12.47	<0.001	0.024
Medial and inferior temporal gyri right (posterior part)	4072.61 (26.97)	3732.66 (29.26)	71.66	<0.001	0.123
Medial and inferior temporal gyri left (posterior part)	4177.71 (28.76)	3840.23 (31.20)	62.10	<0.001	0.109

Superior temporal gyrus right (posterior part)	1302.68 (11.01)	1228.80 (11.95)	20.30	<0.001	0.038
Superior temporal gyrus left (posterior part)	1152.91 (10.09)	1083.83 (10.95)	21.14	<0.001	0.040
Cingulate gyrus right (anterior part)	1547.01 (12.99)	1431.32 (14.10)	35.74	<0.001	0.066
Cingulate gyrus left (anterior part)	1362.69 (13.28)	1294.44 (14.41)	11.91	<0.001	0.023
Cingulate gyrus right (posterior part)	1788.53 (14.59)	1650.82 (15.83)	40.18	<0.001	0.073
Cingulate gyrus left (posterior part)	1749.50 (13.78)	1639.70 (14.96)	28.61	<0.001	0.053
Frontal lobe right	26697.21 (151.57)	24731.87 (164.45)	75.82	<0.001	0.130
Frontal lobe left	26541.42 (150.72)	24703.84 (163.54)	67.03	<0.001	0.116
Parietal lobe right	18865.28 (101.82)	17910.62 (110.47)	39.64	<0.001	0.072
Parietal lobe left	19011.73 (103.08)	18018.39 (111.84)	41.88	<0.001	0.076

Caudate nucleus right	1991.69 (13.72)	1898.66 (14.89)	20.73	<0.001	0.039
Caudate nucleus left	1935.77 (13.84)	1853.34 (15.01)	16.00	<0.001	0.030
Thalamus right	4254.28 (18.42)	4091.43 (19.98)	35.25	<0.001	0.065
Thalamus left	4171.84 (17.80)	4001.43 (19.32)	41.31	<0.001	0.075
Subthalamic nucleus right	224.48 (1.26)	210.91 (1.37)	52.09	<0.001	0.093
Subthalamic nucleus left	187.28 (1.16)	176.84 (1.26)	36.51	<0.001	0.067
Lentiform nucleus right	3235.56 (18.25)	3058.51 (19.80)	42.43	<0.001	0.077
Lentiform nucleus left	3432.98 (18.12)	3244.57 (19.66)	48.74	<0.001	0.087
Corpus Callosum	2999.20 (23.55)	2871.10 (25.55)	13.34	<0.001	0.026

All analyses are controlled for postconceptional age at scan and birth weight. All *p* values are FDR-corrected for multiple comparisons. Means values are estimated marginalised means corrected for birth weight and postconceptional age at birth.

Table S6. Sex-by-age interactions

**Region**	**F**	p_{FDR}	η_p^2	**Direction**
Total Brain Volume	0.85	0.427	0.00	Male>Female
Total Intracranial Volume	2.50	0.344	0.01	Male>Female
Cerebrospinal Fluid	7.24	0.044	0.01	Male>Female
Total Cortical Gray Matter	1.76	0.370	0.00	Male>Female
Total Subcortical Gray Matter	1.04	0.427	0.00	Male>Female
Total White Matter	0.15	0.670	0.00	Male>Female
Anterior temporal lobe lateral part left	0.50	0.553	0.001	Male>Female
Anterior temporal lobe lateral part right	1.24	0.324	0.002	Female>Male
Parahippocampal gyrus left (anterior part)	0.05	0.858	0.000	Male>Female

Parahippocampal gyrus right (anterior part)	0.00	0.993	0.000	Female>Male
Superior temporal gyrus left (middle part)	0.00	0.972	0.000	Male>Female
Superior temporal gyrus right (middle part)	0.17	0.737	0.000	Female>Male
Medial and inferior temporal gyri left (anterior part)	1.82	0.228	0.003	Male>Female
Medial and inferior temporal gyri right (anterior part)	2.04	0.204	0.004	Male>Female
Lateral occipitotemporal gyrus left (anterior part)	0.00	0.993	0.000	Female>Male
Lateral occipitotemporal gyrus right (anterior part)	0.42	0.589	0.001	Male>Female

Cerebellum left	0.33	0.639	0.001	Male>Female
Cerebellum right	0.51	0.551	0.001	Male>Female
Brainstem	1.34	0.305	0.002	Male>Female
Insula right	1.91	0.217	0.004	Male>Female
Insula left	4.21	0.059	0.008	Male>Female
Occipital lobe right	2.07	0.201	0.004	Male>Female
Occipital lobe left	0.01	0.954	0.000	Male>Female
Parahippocampal gyri right (posterior part)	0.23	0.693	0.000	Male>Female
Parahippocampal gyri rleft (posterior part)	0.32	0.643	0.001	Female>Male
Latetal occipitotemporal	0.31	0.643	0.001	Male>Female

gyus right (posterior part)				
Lateral occipitotemporal gyrus left (posterior part)	0.30	0.644	0.001	Male>Female
Medial and inferior temporal gyri right (posterior part)	1.02	0.374	0.002	Male>Female
Medial and inferior temporal gyri left (posterior part)	2.41	0.166	0.005	Male>Female
Superior temporal gyrus right (posterior part)	3.81	0.074	0.007	Male>Female
Superior temporal gyrus left (posterior part)	8.31	0.006	0.020	Male>Female
Cingulate gyrus right (anterior part)	0.04	0.875	0.000	Female>Male
Cingulate gyrus left (anterior part)	5.75	0.025	0.01	Female>Male
Cingulate gyrus right (posterior part)	1.78	0.232	0.003	Male>Female

Cingulate gyrus left (posterior part)	0.16	0.745	0.000	Male>Female
Frontal lobe right	2.52	0.156	0.005	Male>Female
Frontal lobe left	2.59	0.151	0.005	Male>Female
Parietal lobe right	1.49	0.277	0.003	Male>Female
Parietal lobe left	2.22	0.184	0.004	Male>Female
Caudate nucleus right	0.05	0.858	0.000	Female>Male
Caudate nucleus left	0.07	0.840	0.000	Female>Male
Thalamus right	2.72	0.142	0.005	Male>Female
Thalamus left	1.67	0.248	0.003	Male>Female
Subthalamic nucleus right	1.07	0.363	0.002	Male>Female

Subthalamic nucleus left	0.12	0.783	0.000	Female>Male
Lentiform nucleus right	1.10	0.357	0.002	Male>Female
Lentiform nucleus left	1.59	0.260	0.003	Male>Female
Corpus Callosum	2.60	0.151	0.005	Male>Female

All *p* values are FDR-corrected for multiple comparisons.

Table S7. *Regional sex-by-age interactions controlling for total brain volume*

**Region**	**F**	p_{FDR}	η_p^2	**Direction**
Hippocampus left	1.48	0.330	0.003	Male>Female
Hippocampus right	1.11	0.398	0.002	Male>Female
Amygdala left	0.24	0.690	0.000	Male>Female
Amygdala right	0.01	0.942	0.000	Female>Male
Anterior temporal lobe medial part left	0.14	0.766	0.000	Male>Female

Anterior temporal lobe medial part right	0.61	0.527	0.001	Female>Male
Anterior temporal lobe lateral part left	0.05	0.864	0.000	Male>Female
Anterior temporal lobe lateral part right	4.29	0.073	0.008	Female>Male
Parahippocampal gyrus left (anterior part)	0.14	0.766	0.000	Female>Male
Parahippocampal gyrus right (anterior part)	0.55	0.549	0.001	Female>Male
Superior temporal gyrus left (middle part)	0.52	0.557	0.001	Female>Male
Superior temporal gyrus right (middle part)	2.09	0.232	0.004	Female>Male
Medial and inferior temporal gyri left (anterior part)	0.97	0.423	0.002	Male>Female

Medial and inferior temporal gyri right (anterior part)	1.20	0.374	0.002	Male>Female
Lateral occipitotemporal gyrus left (anterior part)	0.41	0.598	0.001	Female>Male
Lateral occipitotemporal gyrus right (anterior part)	0.02	0.921	0.000	Male>Female
Cerebellum left	0.01	0.942	0.000	Female>Male
Cerebellum right	0.02	0.929	0.000	Male>Female
Brainstem	0.49	0.570	0.001	Male>Female
Insula right	1.06	0.399	0.002	Male>Female
Insula left	4.07	0.082	0.008	Male>Female
Occipital lobe right	1.29	0.365	0.003	Male>Female

Occipital lobe left	1.09	0.398	0.002	Female>Male
Parahippocampal gyri right (posterior part)	0.02	0.929	0.000	Female>Male
Parahippocampal gyri rleft (posterior part)	1.56	0.317	0.003	Female>Male
Latetal occipitotemporal gyus right (posterior part)	0.01	0.942	0.000	Male>Female
Lateral occipitotemporal gyrus left (posterior part)	0.01	0.942	0.000	Male>Female
Medial and inferior temporal gyri right (posterior part)	0.21	0.713	0.000	Male>Female
Medial and inferior temporal gyri left (posterior part)	1.73	0.285	0.003	Male>Female
Superior temporal gyrus right (posterior part)	3.04	0.141	0.006	Male>Female

Superior temporal gyrus left (posterior part)	8.03	0.011	0.016	Male>Female
Cingulate gyrus right (anterior part)	1.25	0.368	0.000	Female>Male
Cingulate gyrus left (anterior part)	14.19	0.000	0.027	Female>Male
Cingulate gyrus right (posterior part)	0.95	0.425	0.002	Male>Female
Cingulate gyrus left (posterior part)	0.21	0.710	0.000	Female>Male
Frontal lobe right	3.72	0.098	0.007	Male>Female
Frontal lobe left	4.07	0.082	0.008	Male>Female
Parietal lobe right	0.75	0.484	0.001	Male>Female
Parietal lobe left	2.21	0.222	0.004	Male>Female

Caudate nucleus right	1.25	0.368	0.002	Female>Male
Caudate nucleus left	1.45	0.333	0.003	Female>Male
Thalamus right	2.20	0.222	0.004	Male>Female
Thalamus left	0.84	0.460	0.002	Male>Female
Subthalamic nucleus right	0.34	0.639	0.001	Male>Female
Subthalamic nucleus left	0.98	0.422	0.002	Female>Male
Lentiform nucleus right	0.32	0.642	0.001	Male>Female
Lentiform nucleus left	0.74	0.486	0.001	Male>Female
Corpus Callosum	1.76	0.283	0.003	Male>Female

All analyses are controlled for total brain volume. All *p* values are FDR-corrected for multiple comparisons.

Table S8. *Regional sex-by-age interactions controlling for intracranial volume*

Region	F	p_{FDR}	η_p^2
Hippocampus left	0.57	0.604	0.001
Hippocampus right	0.33	0.715	0.001
Amygdala left	0.00	0.989	0.000
Amygdala right	0.33	0.715	0.000
Anterior temporal lobe medial part left	0.00	0.989	0.000
Anterior temporal lobe medial part right	1.67	0.301	0.000
Anterior temporal lobe lateral part left	0.02	0.937	0.000
Anterior temporal lobe lateral part right	6.14	0.028	0.000
Parahippocampal gyrus left (anterior part)	0.62	0.587	0.001
Parahippocampal gyrus right (anterior part)	1.39	0.359	0.003

Superior temporal gyrus left (middle part)	1.50	0.334	0.003
Superior temporal gyrus right (middle part)	3.50	0.113	0.007
Medial and inferior temporal gyri left (anterior part)	0.16	0.803	0.000
Medial and inferior temporal gyri right (anterior part)	0.20	0.788	0.000
Lateral occipitotemporal gyrus left (anterior part)	0.96	0.465	0.000
Lateral occipitotemporal gyrus right (anterior part)	0.04	0.903	0.000
Cerebellum left	0.47	0.649	0.001
Cerebellum right	0.18	0.797	0.000
Brainstem	0.00	0.975	0.000
Insula right	0.16	0.803	0.000
Insula left	1.75	0.295	0.003

Occipital lobe right	0.14	0.820	0.000
Occipital lobe left	3.44	0.116	0.007
Parahippocampal gyri right (posterior part)	0.40	0.682	0.001
Parahippocampal gyri rleft (posterior part)	2.78	0.169	0.005
Latetal occipitotemporal gyus right (posterior part)	0.11	0.836	0.000
Lateral occipitotemporal gyrus left (posterior part)	0.09	0.865	0.000
Medial and inferior temporal gyri right (posterior part)	0.13	0.822	0.000
Medial and inferior temporal gyri left (posterior part)	0.31	0.725	0.001
Superior temporal gyrus right (posterior part)	1.63	0.307	0.000
Superior temporal gyrus left (posterior part)	5.79	0.033	0.000

Cingulate gyrus right (anterior part)	2.29	0.220	0.004
Cingulate gyrus left (anterior part)	15.97	0.000	0.030
Cingulate gyrus right (posterior part)	0.07	0.873	0.000
Cingulate gyrus left (posterior part)	1.22	0.391	0.002
Frontal lobe right	0.13	0.822	0.000
Frontal lobe left	0.17	0.803	0.000
Parietal lobe right	0.08	0.873	0.000
Parietal lobe left	0.05	0.903	0.000
Caudate nucleus right	2.22	0.229	0.004
Caudate nucleus left	2.46	0.201	0.005
Thalamus right	0.49	0.644	0.000
Thalamus left	0.02	0.940	0.000
Subthalamic nucleus right	0.01	0.958	0.000

Subthalamic nucleus left	1.74	0.295	0.003
Lentiform nucleus right	0.00	0.989	0.000
Lentiform nucleus left	0.09	0.865	0.000
Corpus Callosum	0.50	0.639	0.001

All analyses are controlled for intracranial volume. All p values are FDR-corrected for multiple comparisons.

Figure S1. *Distribution of Postnatal Age at Scan*

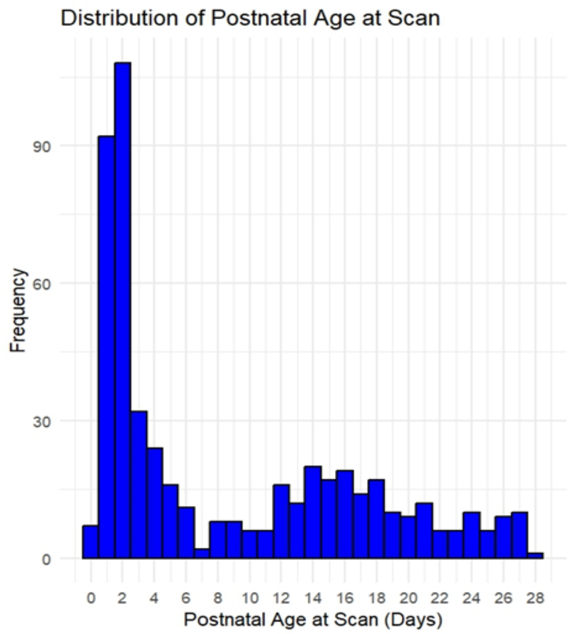

Figure S2. *Regional sex differences by effect size after controlling for intracranial volume*

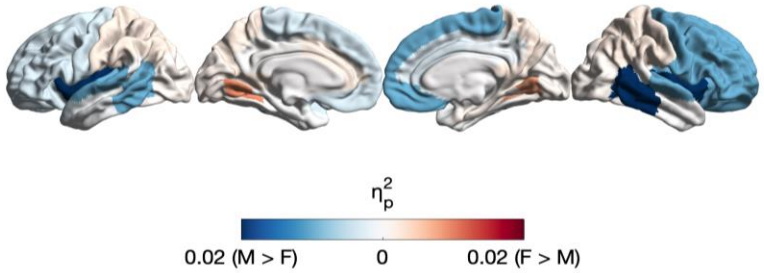

Note. Supplementary Figure 1 depicts $\boldsymbol{\eta_p}^2$ values of regions showing statistically significant sex differences (FDR-corrected $p < 0.05$) after controlling for intracranial volume.
